# Supplementary material for: Employment status before and after open heart valve surgery: A cohort study
Source: PLoS One. 2020 Oct 7;15(10):e0240210. doi: 10.1371/journal.pone.0240210 (PMC7541055; doi:10.1371/journal.pone.0240210)

S1 Fig. Probability distribution of employment status six months before and after surgery among the total population of patients between 18-63 years

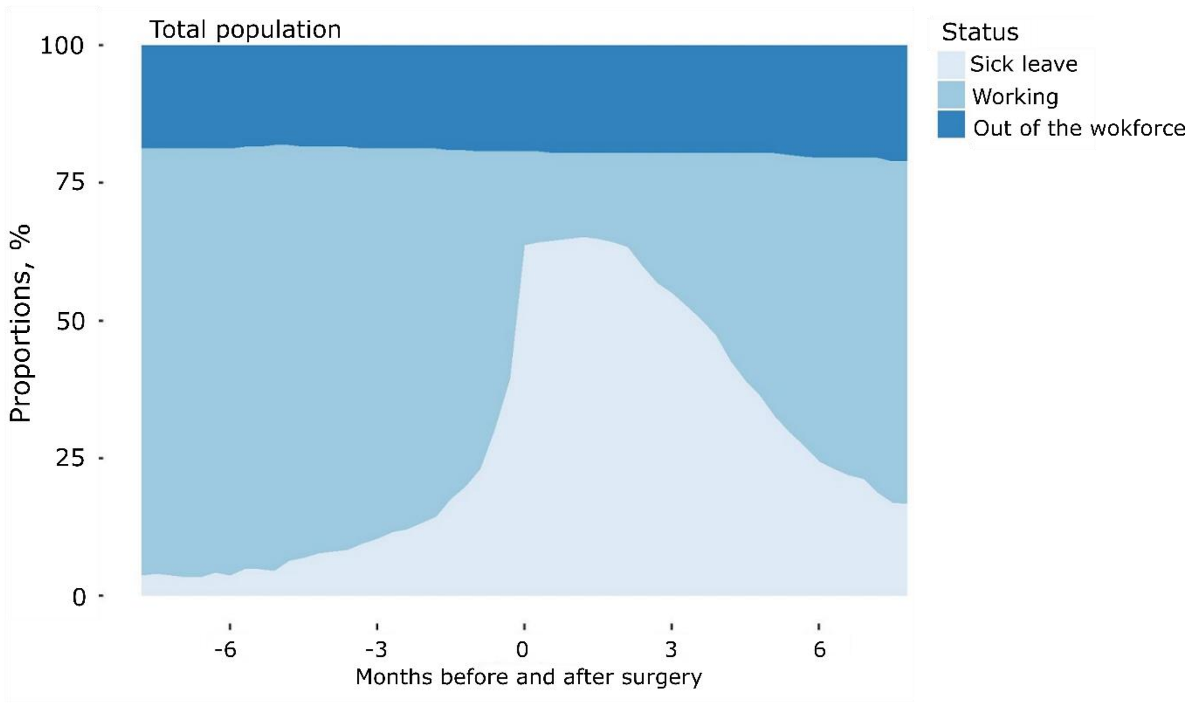

Supplement: S1 Fig — (PDF) [file pone.0240210.s001.pdf]
