# Supplementary figures and images for: Employment status before and after open heart valve surgery: A cohort study
Source: PLoS One. 2020 Oct 7;15(10):e0240210. doi: 10.1371/journal.pone.0240210 (PMC7541055; doi:10.1371/journal.pone.0240210)

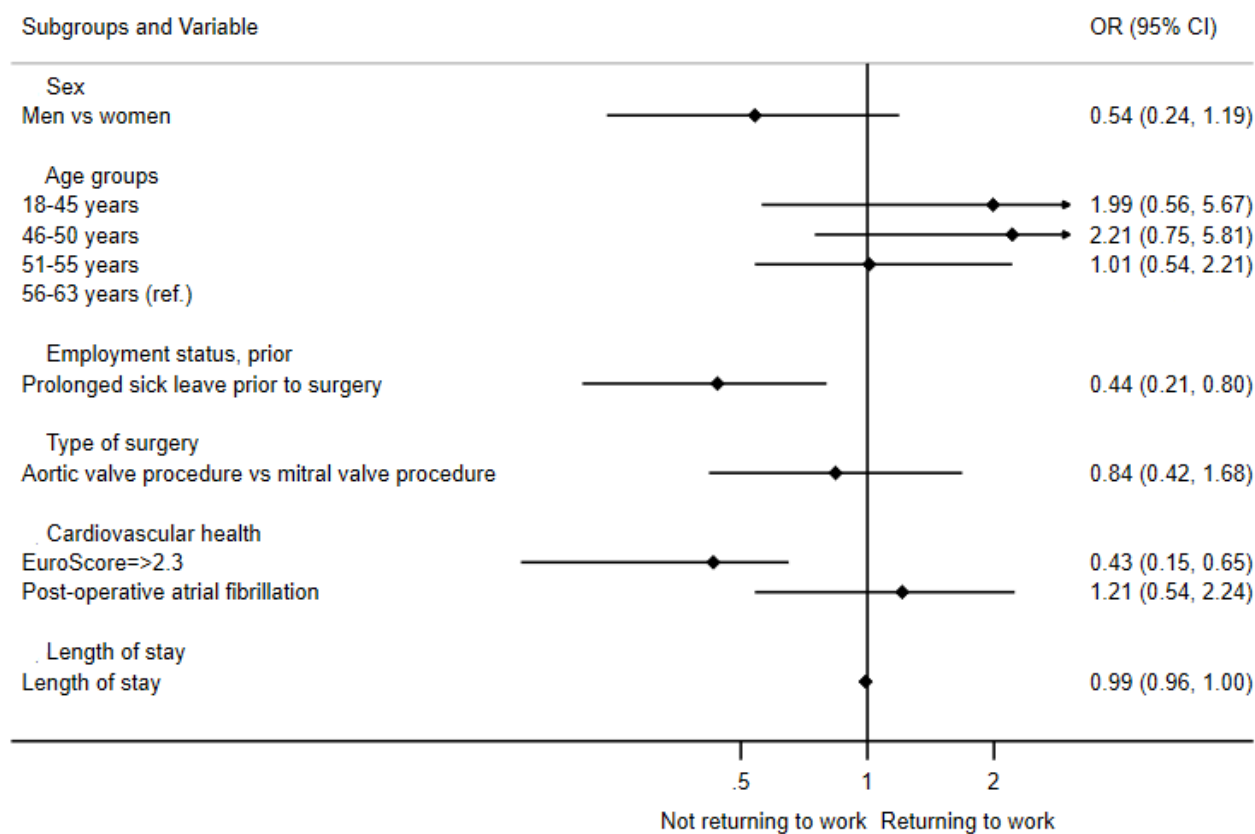

Supplement: S2 Fig — (PDF) [file pone.0240210.s002.pdf]
