# Supplementary material for: Employment status before and after open heart valve surgery: A cohort study
Source: PLoS One. 2020 Oct 7;15(10):e0240210. doi: 10.1371/journal.pone.0240210 (PMC7541055; doi:10.1371/journal.pone.0240210)
Supplement: S1 Table — (PDF) [file pone.0240210.s003.pdf]

S1 Table. Baseline characteristics of the total population (including patients on early retirement)

	**Total population* (n=347)**
**Characteristics**	
Sex, male, n (%)	267 (77)
Age-groups, n (%)	
18-45 years	50 (14)
46-50 years	55 (16)
51-55 years	64 (18)
56-63 years	178 (51)
Living alone, n (%)	94 (27)
**Pre-operative information**	
Reduced pulmonary function[a], n (%)	117 (34)
EuroScore II (logistic), median (IQR)	1.24 (0.75-2.48)
EuroScoreII ≥2.3, n (%)	95 (27)
Estimated glomerular filtration rate ml/min.[b], median (IQR)	101 (82-121)
Atrial fibrillation, n (%)	58 (17)
Diabetes[c], n (%)	30 (9)
Ejection fraction ≤50%, n (%)	97 (28)
NYHA class ≥3, n (%)	113 (33)
Body Mass Index, median (IQR)	26 (24-30)
Current or former smoker, n (%)	182 (52)
Alcohol intake above national recommendations, n (%)	39 (11)
Primary diagnosis, n (%)	
Aortic valve stenosis	146 (42)
Aortic valve regurgitation	97 (28)
Mitral valve stenosis	<5 (1)
Mitral valve regurgitation	98 (29)
**Surgical information, n (%)**	
Type of valve procedure[d]	
Aortic valve, biological	53 (15)
Aortic valve, mechanical	182 (52)
Aortic valve, repair	10 (3)
Mitral valve, replacement[e]	30 (9)
Mitral valve, repair	70 (20)
Concomitant CABG	36 (10)
**Post-procedure related, n (%)**	
Re-operation	23 (7)
Prolonged length of stay[f], intensive care unit	35 (10)
Post-operative atrial fibrillation	165 (48)
New onset post-operative atrial fibrillation	124 (36)
Length of stay	
4-7 days	120 (35)
8-12 days	150 (43)
≥13 days	77 (22)

IQR, interquartile range, 25th to 75th quartile. NYHA, New York Heart Association Class

* The total population of patients between 18-63 years

[a] Patients with forced expiratory volume,% ≤80% of predicted value and / or a history of chronic obstructive pulmonary disease

[b] Estimated glomerular filtration rate estimated by the Cockcroft-Gault Equation

[c] Patients with diabetes; insulin, per oral and non-pharmacological treatment

[d] One patient had surgery on the tricuspidal valve and are not shown in the table, but included in the analyses

[e] Both biological and mechanical mitral valve replacement

[f] Admission at intensive care unit for more than one day
