# Supplementary material for: Employment status before and after open heart valve surgery: A cohort study
Source: PLoS One. 2020 Oct 7;15(10):e0240210. doi: 10.1371/journal.pone.0240210 (PMC7541055; doi:10.1371/journal.pone.0240210)
Supplement: S2 Table — (PDF) [file pone.0240210.s004.pdf]

S2 Table. Patterns of sick leave and return to the workforce among the population being part of the workforce and divided by age groups

	All patients*	***Age groups***			
	(n=282)	18-45 years (n=42)	45-50 years (n=46)	51-55 years (n=55)	56-63 years (n=139)
Status "sick leave" at specific time points, n (%)					
6 months before surgery	7 (2)	2 (5)	1 (2)	0 (0)	4 (3)
3 months before surgery	29 (10)	4 (10)	4 (9)	2 (4)	19 (14)
2 months before surgery	42 (15)	5 (12)	5 (11)	6 (11)	26 (19)
1 month before surgery	69 (24)	8 (12)	9 (13)	12 (17)	40 (58)
Time of surgery	223 (79)	28 (67)	36 (78)	41 (75)	118 (85)
1 month after surgery	226 (80)	29 (69)	36 (78)	44 (80)	117 (84)
2 months after surgery	208 (74)	27 (64)	35 (76)	41 (75)	105 (76)
3 months after surgery	174 (62)	23 (55)	29 (63)	35 (64)	87 (63)
6 months after surgery	58 (21)	12 (29)	6 (13)	12 (22)	28 (20)

*Among all patients being part of the workforce before surgery.
