# Supplementary material for: Employment status before and after open heart valve surgery: A cohort study
Source: PLoS One. 2020 Oct 7;15(10):e0240210. doi: 10.1371/journal.pone.0240210 (PMC7541055; doi:10.1371/journal.pone.0240210)
Supplement: S3 Table — (PDF) [file pone.0240210.s005.pdf]

S3 Table. Patterns of employment status before and after open heart valve surgery.

		Sick leave	***Working*** ***(not receiving paid sick leave benefit)***	***Out of the workforce***
n=347		n (% / %)[a]	n (% / %)[a]	n (%)[b]
Before surgery	6 months	7 (2/2)	277 (80/98)	63 (18)
	3 months	29 (8/10)	254 (73/90)	64 (18)
	2 months	42 (12/15)	240 (69/85)	65 (19)
	1 month	69 (20/24)	213 (61/76)	65 (19)
Time of surgery	0	223 (64/79)	59 (17/21)	65 (19)
After surgery	1 month	226 (65/80)	53 (15/19)	68 (20)
	2 months	208 (60/74)	71 (20/25)	68 (20)
	3 months	174 (50/62)	105 (30/37)	68 (20)
	6 months	58 (17/21)	219 (63/77)	71 (20)

[a] % of the total population / % of patients in the workforce before surgery

[b] % of the total population
